# Left Behind: The Unmet Need for Breast Cancer Research in Mississippi

**DOI:** 10.3390/cancers17101652

**Published:** 2025-05-13

**Authors:** Rifath Ara Alam Barsha, Jasmine Miller-Kleinhenz

**Affiliations:** John D. Bower School of Population Health, University of Mississippi Medical Center, 2500 N State Street, Jackson, MS 39216, USA; rbarsha@umc.edu

**Keywords:** breast cancer, Mississippi, disparity

## Abstract

**Background/Objective:** Mississippi has the highest breast cancer mortality rate in the nation, yet there remains a limited understanding of the factors currently contributing to breast cancer in the state. This systematic review aims to provide insights into breast cancer epidemiology, disparities, and outcomes in Mississippi by synthesizing existing research published over the past 25 years. **Methods:** A systematic search of PubMed and Google Scholar identified studies published between 2000 and 2024 that focused on breast cancer in Mississippi. Quality appraisal was performed using the Joanna Briggs Institute (JBI) checklist, and a narrative synthesis approach was used to analyze the findings. **Results:** The initial literature search yielded 33 articles. After removing duplicates, screening titles and abstracts, and conducting a full-text review, 15 studies met the inclusion criteria. The findings revealed significant racial and geographic disparities in breast cancer incidence, mortality, and access to care. Black women in Mississippi are more likely to be diagnosed in later stages and experience worse outcomes, primarily due to socioeconomic disadvantages, healthcare access barriers, and structural inequities. Geographic barriers, particularly in rural areas, further worsen these disparities. **Conclusions:** This review identified a scarcity of breast cancer research in Mississippi over the last 25 years, with only three studies in the past 5 years, leaving critical knowledge gaps in understanding current dynamics. This review highlights an urgent need for expanded, Mississippi-specific breast cancer research to better understand the factors driving disparities and to develop culturally tailored, evidence-based interventions to address breast cancer disparities.

## Simple Summary

Mississippi has the highest death rate from breast cancer in the United States, yet major gaps remain in understanding the factors currently contributing to breast cancer in the state. This study aimed to provide valuable insights into breast cancer epidemiology, disparities, and outcomes within Mississippi. We conducted a comprehensive review of all available research on breast cancer in Mississippi from the past 25 years. Our findings indicate that many women, especially Black women, are diagnosed in later stages when the disease is more difficult to treat. Barriers such as cost, lack of health insurance, and long travel distances make it harder for many women to receive timely screening and care. Despite these challenges and the state’s high death rate, research on breast cancer in Mississippi remains limited, with only 15 studies published over the last 25 years. Our findings highlight the urgent need for more state-specific research to understand the factors contributing to breast cancer disparities in Mississippi and to develop effective strategies to reduce breast cancer deaths and ensure that all women have access to early detection and high-quality care.

## 1. Introduction

Breast cancer is the most commonly diagnosed cancer among women after skin cancer and remains one of the leading causes of cancer-related deaths in the United States (US) [1]. The incidence of breast cancer has shown an upward trend from 2012 to 2021, with an annual increase of 1%, primarily driven by localized-stage and hormone-receptor-positive cases [2]. On the other hand, the breast cancer mortality rate decreased steadily by 44% between 1989 and 2022, largely due to advancements in cancer screening and treatment modalities [2]. Despite this decline in mortality over recent decades, breast cancer remains the second most common cause of cancer-related death among US women, and among Black and Hispanic women, it is the leading cause of cancer-related death [1]. In addition, disparities in breast cancer outcomes persist across racial, socioeconomic, and geographic lines. Black women, for example, are more likely to be diagnosed with late-stage disease and experience higher mortality rates compared to their White counterparts [3]. Despite having a 5% lower incidence of breast cancer, Black women experience a 38% higher mortality rate than White women [1]. While the higher prevalence of triple-negative breast cancer (TNBC) contributes to this disparity, it does not fully explain the survival gap. Black women have the lowest survival rates across nearly all breast cancer subtypes and stages, except for localized disease, highlighting the disadvantages and impact of social determinants of health [1]. These disparities are particularly pronounced in states such as Mississippi, which consistently reports the highest breast cancer mortality rates in the US.

Mississippi has the highest breast cancer mortality rate in the nation, at 23.4 per 100,000, compared to the national average of 19.3 per 100,000 [4]. While the national breast cancer incidence rate has generally been lower among Black women than White women in recent years, Mississippi presents a different pattern, with higher incidence rates among Black women compared to their White counterparts [5]. The age-adjusted incidence rate of breast cancer for the years 2017–2021 among Black women was 158.61 per 100,000, while for White women, it was 145.91 per 100,000. This disparity is even more pronounced in mortality rates: Black women in Mississippi experienced a breast cancer mortality rate of 30.9 per 100,000 in 2021, compared to 21.6 per 100,000 for White women. Additionally, the breast cancer screening rate among older women in Mississippi is one of the lowest in the nation [6].

Despite having the highest breast cancer mortality rates in the nation, Mississippi remains understudied in breast cancer research, resulting in critical knowledge gaps in understanding the current landscape and addressing this pressing issue. While national studies highlighted racial and socioeconomic disparities, they often fail to capture the unique social, economic, and healthcare challenges faced by the local population. Therefore, this systematic review seeks to bridge these gaps by synthesizing existing research over a 25-year period to explore the state-specific dynamics of breast cancer. By summarizing and evaluating published studies, this review aims to provide valuable insights into breast cancer epidemiology, disparities, and outcomes within Mississippi. These findings will inform the direction of future research, guide targeted interventions, and support policy development aimed at reducing disparities and improving breast cancer outcomes in the state.

## 2. Materials and Methods

### 2.1. Study Design and Eligibility Criteria

We conducted a systematic literature review from December 2024 to January 2025. The inclusion criteria for this study were studies focused on breast cancer in Mississippi and published between 1 January 2000 and 31 December 2024. This time period was selected for the literature search to capture research published over the past 25 years and to provide a comprehensive overview of breast cancer research in Mississippi. Conference abstracts, dissertations, non-peer-reviewed papers, opinion pieces not published in peer-reviewed journals, and studies that did not specify Mississippi as the study location or did not include Mississippi-specific data were excluded. This approach ensured the inclusion of relevant, high-quality studies while excluding sources that lacked rigor or geographic specificity.

### 2.2. Search Strategy

A comprehensive literature search was conducted using two electronic databases: PubMed (https://pubmed.ncbi.nlm.nih.gov/, accessed on 3 December 2024) and Google Scholar (https://scholar.google.com/, accessed on 3 December 2024). The search strategy involved combinations of the following keywords: “breast cancer”, “breast tumor”, “Mississippi”, and “women”. Individual keywords and their combinations were entered using AND and OR operators. We combined Mississippi-specific search terms with “neoplasms” OR “cancer” to identify articles focused on multiple cancer sites, including breast cancer. The specified timeframe was consistently applied across all databases. The bibliographic references from the identified publications were manually searched to identify additional relevant papers.

### 2.3. Study Selection

**Figure 1:**
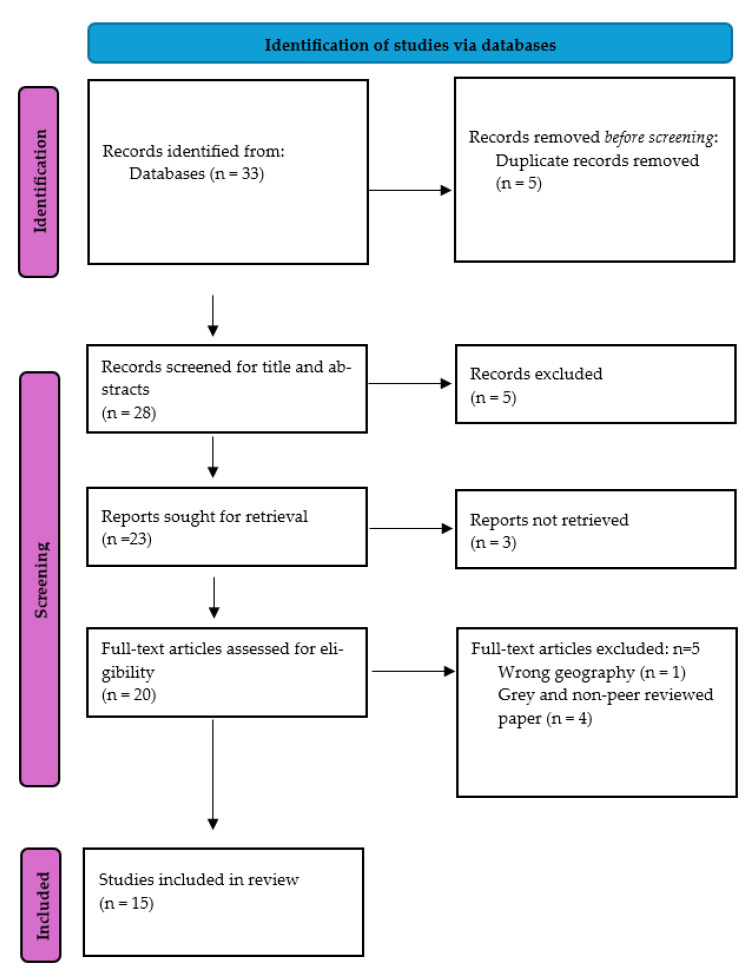
Flow diagram of review process according to Preferred Reporting Items for Systematic Reviews and Meta-Analysis (PRISMA).

All identified articles were imported into EndNote version 21 (developed by Clarivate) to remove duplicates [7]. Title and abstract screening for eligibility were conducted by one investigator (R.A.A.B.) and reviewed for accuracy by another investigator (J.M.-K.). For studies requiring clarification, problems or disagreements were resolved through discussion between the investigators. Following the initial screening, a full-text review of eligible studies was conducted. The results of this process are presented using a Preferred Reporting Items for Systematic Reviews and Meta-Analysis (PRISMA) flowchart [8] (Figure 1).

### 2.4. Data Extraction and Quality Appraisal

We extracted data into a pre-designed data extraction form. The extracted data included the following details about the study, including the first author’s name, year of publication, geography, study aim, design, outcomes, and key findings. Given that this systematic review included articles with diverse study designs, we used the Joanna Briggs Institute (JBI) checklists for respective study designs to conduct our quality appraisal [9].

### 2.5. Data Synthesis

The data extracted from the included studies were analyzed using a narrative synthesis approach. This method involved organizing and summarizing findings to identify patterns, themes, and relationships across the studies. The synthesis focused on key aspects such as population characteristics, study design, outcomes, and disparities in breast cancer epidemiology within Mississippi.

## 3. Results

### 3.1. Study Characteristics

The database search identified 33 studies, and 5 duplicate studies were removed before screening. After title and abstract screening, an additional five studies were excluded due to irrelevance. A total of 23 studies were sought for full-text retrieval, but full-text access was unavailable for 3 studies. A full-text review was conducted on 20 studies based on the study eligibility criteria, resulting in the exclusion of additional studies. Ultimately, 15 articles were included in the review.

The included studies utilized various designs, including cross-sectional studies (n = 11), retrospective cohort studies (n = 2), a quasi-experimental study (n = 1), and an opinion piece (n = 1). The characteristics of these studies are detailed in Table 1.

### 3.2. Epidemiology of Breast Cancer in Mississippi

**Figure 2:**
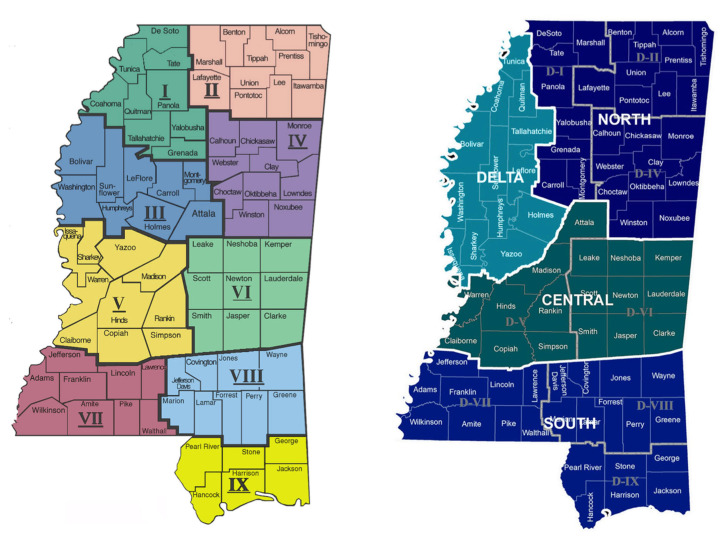
Public health districts in Mississippi (map images are from the Mississippi Department of Health [25,26]).

Seven studies published between 2004 and 2023 reported breast cancer statistics and highlighted disparities in breast cancer outcomes in Mississippi [11,12,15,16,19,20,24]. Of these seven studies, five were solely focused on Mississippi, and the other two explored state variations and reported limited data on Mississippi. DeSantis et al. found that from 1975 to 2004, the breast cancer mortality rate among Black women in Mississippi increased by 1.8% per year, while the rate among White women decreased by 1.0% per year since 1989 [12]. Smith et al. investigated breast cancer incidence across different health districts in Mississippi. Mississippi is divided into nine health districts (Districts I to IX), with the Delta region primarily encompassing Health Districts I and III (Figure 2). The study found that Black women in Districts VII and III had significantly higher incidence and mortality rates compared to the other seven health districts [20]. Additionally, District III had a significantly higher proportion of individuals with less than a high school education. The age at diagnosis was lowest in District V, where individuals were diagnosed at an earlier age. Nichols et al. (2014) found a moderate correlation between breast cancer mortality-to-incidence ratios and the percentage of the population living below the federal poverty level [16].

One study examined the relationship between environmental chemicals and breast cancer incidence in Mississippi counties [11]. Environmental chemicals examined in this study included carbon monoxide (CO), sulfur dioxide (SO_2_), nitrogen oxides, particulate matter (PM_10_ and PM_2.5_), volatile organic compounds (VOCs), and ammonia (NH_3_). The study identified counties with a higher breast cancer incidence compared to the state’s median rate in 1998. Six counties, Yazoo, Copiah, George, Forrest, Stone, and Hinds, had incidence rates 40% or higher than the state average. Additionally, Noxubee, Jefferson, Jones, Perry, Scott, Chickasaw, Madison, Yalobusha, Clay, Tishomingo, and Warren had higher rates than the state median. Harrison, Hinds, Jackson, Forrest, Rankin, Jones, Lauderdale, Perry, DeSoto, and Scott counties had the highest levels of maximum chemical emissions, with amounts 60% or higher than the state’s median emissions. However, the study could not report individual chemicals, due to the use of aggregated data on chemical emissions from multiple sources. Breast cancer incidence was significantly correlated with ammonia levels, as well as the minimum and maximum emissions from facilities within these counties. The highest-emitting sources included industries such as inorganic pigments, natural gas transmission, electrical services, paper mills, sawmills, and planning mills, general medical and surgical hospitals, petroleum refining, and shipbuilding and repair.

Significant differences exist in the stage of breast cancer diagnosis among racial groups in Mississippi. Studies highlighted that Black women in the state were disproportionately diagnosed in later stages of breast cancer compared to White women [15,19,20]. Fortune et al. (2017) reported that approximately 44% of Black women were diagnosed in a late stage of breast cancer, compared to 30% of White women. Additionally, uninsured women were more likely to be diagnosed in a later stage of the disease [19]. Another study reported substantial racial disparities in TNBC incidence across states, showing that Black women in Mississippi, Delaware, Missouri, and Louisiana had the highest rates among all states and racial groups [24].

Geography also influenced breast cancer diagnosis. Women living in rural Mississippi were more likely to present with advanced-stage breast cancer compared to in situ or localized breast cancer, with rates of 4% for White women and 19% for Black women. Black women residing in urban Mississippi had 25% higher odds of being diagnosed in a later stage. In comparison, rural Black women had 47% higher odds compared to their urban and rural White counterparts, respectively [15].

### 3.3. Cancer Screening, Access to Care, and Programs

Nine studies examined cancer screening, access to care, and related programs [10,13,14,16,17,18,21,22,23].

Access to mammography services is a critical factor in early breast cancer detection and improved outcomes. Zahad et al. (2019) identified clusters of limited access to mammography services in parts of the Mississippi Delta [21]. The utilization of mammography services also differs between Black and White women in Mississippi. According to Nichols et al. (2014), among women aged 40 and older, 73.13% of Black women had undergone mammography and clinical breast exams, compared to 82.57% of White women [16]. The authors also reported that in Mississippi, 84.10% of women aged 40 and older live within a 30 min drive of a mammography facility. In comparison, the percentages are higher in neighboring states: 94.36% in Louisiana, 94.50% in Tennessee, and 93.04% in Alabama [16].

Another study by Mayfield-Johnson et al. (2016) identified multiple barriers to breast cancer screening among participants, with the majority citing cost and limited access as obstacles to mammography [18]. Additionally, lack of health insurance was a frequently reported barrier. The researchers utilized community-based approaches, including partnerships with organizations and outreach efforts led by community health workers, to recruit participants and increase screening rates. Their findings emphasized the importance of effective outreach and collaboration in overcoming barriers to mammography screening. Mayfield-Johnson et al. also found that nearly half of the study participants aged 40 years or older, who were recommended to have a mammogram according to clinical guidelines, had never undergone the procedure. Additionally, about one-third of the participants reported having a mammogram more than two years ago, despite most of them being aware of the screening recommendations. Furthermore, one-third of the participants were not engaged in monthly self-breast examinations [18]. The study by Higginbotham et al. (2001) found that rural residents in Mississippi, particularly rural Black women, face significant barriers to accessing and utilizing early cancer detection programs and quality medical care [10]. The article by Houston (2013) also stated the alarming rate of breast cancer among women in Mississippi and emphasized the need to strictly follow the American Cancer Society guidelines for early detection [13].

Efforts to improve breast cancer screening and early detection in Mississippi have included programs aimed at reducing disparities in access to care. Fortune (2015) analyzed the impact of the Mississippi Breast and Cervical Cancer Program (BCCP) on the stage of breast cancer diagnosis among women enrolled in the program [17]. The Mississippi BCCP was established in July 1998 under the National Breast and Cervical Cancer Early Detection Program (NBCCEDP), which Congress authorized through the Breast and Cervical Cancer Mortality Prevention Act of 1990 (Public Health Law 101-354). The NBCCEDP was designed to reduce breast and cervical cancer morbidity and mortality through education and early detection. The Mississippi BCCP aims to serve women who are uninsured or underinsured, medically underserved, living below the federal poverty level, elderly, and from minority populations [17]. The study analyzed data on diagnosed breast cancer cases through the BCCP from 1999 to 2009 and found no significant reduction in late-stage breast cancer diagnoses. While increased screenings led to more breast cancer detections, women in the BCCP were still frequently diagnosed in later stages of the disease. The authors concluded that disparities in late-stage diagnosis persist even with healthcare coverage and emphasized the need for future studies to examine the health-seeking behavior of the population being screened [17].

Some community-based initiatives have also been implemented to improve access to breast cancer screening and care. Wilson-Anderson et al. (2013) conducted a study in two rural Mississippi counties, and they found that rural Black women were receptive to primary health education on breast cancer and showed a certain level of adherence to recommended screenings [14]. One participant who was diagnosed with breast cancer during screening received treatment and became cancer-free after two years, demonstrating the potential positive outcomes of early detection. Additionally, the study documented that community members can be effectively trained to educate others on primary health practices [14]. Williams et al. (2020) conducted a breast cancer screening event during the homecoming festivities of a historically Black university in a rural Mississippi county [22]. During the event, two healthcare providers performed clinical breast exams for 26 Black women and provided tailored risk-reduction counseling. Nearly one-third of the women screened reported never having undergone a breast cancer screening before. The authors concluded that events like this are an effective way to reach women who have never received any form of breast cancer screening, promoting early detection and awareness. Williams (2022) evaluated a cancer screening program in the Jackson Metropolitan Area [23]. A total of 57 women received a mammogram. Participants reported that the program positively influenced their intentions to adopt healthier behaviors, with the majority stating they would perform regular self-breast exams and continue receiving routine mammograms. Evaluation data highlighted that program participants viewed free cancer screenings and the same-day receipt of results as the program’s primary benefits.

### 3.4. Study Quality

The quality assessment is presented in Table 2. Most of the cross-sectional studies were rated highly, with a clearly defined study population, valid measurement tools and criteria, and appropriate statistical analyses [10,12,16,18,20,21,22]. A few studies had an unclear identification of confounding factors or lacked strategies to adjust for their effect [11,15,19,23]. However, according to the checklist, none of the studies were classified as poor quality. Similarly, cohort studies demonstrated strong methodological validity and reliability [17,24]. Despite the absence of a control group, the included quasi-experimental study met other criteria, such as outcome reliability and statistical rigor [14]. Additionally, the opinion piece was credible, providing a well-reasoned, peer-reviewed argument supported by the literature [13].

## 4. Discussion

This systematic review provides a comprehensive understanding of Mississippi breast cancer epidemiology, disparities, and outcomes based on published research over the last 25 years. Surprisingly, we identified very limited research focused on breast cancer in Mississippi. We found only 15 articles that included Mississippi-specific data. Of these, 13 studies focused solely on Mississippi, while the remaining two were national studies that provided limited data on the state. The findings highlight the disproportionate burden of breast cancer and mortality among Black women in the state, as well as the impact of socioeconomic factors and geographic barriers on breast cancer screening and access to care. Additionally, the review emphasizes the importance of state-specific expanded research and targeted interventions to improve early detection and health outcomes.

### 4.1. Racial Disparities

Through this review, we identified several studies documenting racial disparities in breast cancer in Mississippi. A key contributing factor to these disparities is the late-stage diagnosis of breast cancer among Black women, as supported by multiple studies in this review [15,19,20]. Given the higher prevalence of aggressive forms of breast cancer, such as TNBC, among Black women in Mississippi, this disparity is particularly concerning. In addition to possible genetic factors, the Black–White disparities in breast cancer can be partially explained by the evidence documented by other national studies indicating the influence of social determinants of health and structural factors such as socioeconomic status [27], neighborhood segregation [28,29,30], and disproportionate opportunities in Black communities [27] translating into lower access to quality cancer care. While we found two studies that examined the influence of socioeconomic factors such as income [19] and poverty [20], we did not identify any studies that investigated the impact of neighborhood deprivation or other social determinants of health on breast cancer in the context of Mississippi. This gap underscores the need for future research exploring how these factors contribute to breast cancer outcomes and disparities within the state.

### 4.2. Screening and Treatment Access

Limited access to breast cancer screening services has emerged as a critical issue in Mississippi, with studies documenting barriers such as cost, lack of health insurance, and limited availability of mammography facilities [18,21]. Even among women who are aware of breast cancer screening recommendations, adherence remains low. Despite being eligible, many women in Mississippi underutilize mammography services and have longer intervals between screenings [18]. Compared to neighboring states, Mississippi has a lower percentage of residents with access to mammography services within a 30 min drive. When services are available, mammography utilization remains lower among Black women [16]. These findings underscore the potential role of healthcare infrastructure and access-related challenges, as documented in national studies [31,32]. Research indicates that even when Black women self-report similar or higher mammography rates compared to White women, they are more likely to receive screenings at lower-resourced facilities or those lacking accreditation from the American College of Radiology [32]. Additionally, they are more likely to experience delays in follow-up care for abnormal findings [33,34]. As documented by national studies, insurance status also contributes to disparities in access. Black women are less likely than White women to have health insurance [35], which is associated with a higher likelihood of advanced-stage diagnosis and reduced access to quality care [36]. However, regardless of insurance status, Black women are more likely than White women to experience treatment delays, discontinuation of care, and lower adherence to guideline-recommended treatment [37,38,39]. In addition, Mississippi is considered one of the most rural states, with more than half of its residents living in rural areas. Moreover, all or part of Mississippi’s 82 counties are medically underserved. The state’s predominantly rural landscape contributes to an uneven distribution of healthcare resources, significantly impacting residents’ access to care and overall health outcomes [40].

State programs, such as the Mississippi BCCP, were launched to improve access to breast cancer screening services. However, evidence suggests that while the BCCP has increased overall breast cancer detection, it has not significantly reduced late-stage diagnoses. This underscores the need for Mississippi-focused research to identify additional interventions to promote early detection and timely treatment. Community-based initiatives have shown promise in increasing breast cancer screening rates among women in Mississippi [22,23]. These emphasize the importance of community engagement and culturally tailored interventions in addressing screening disparities and improving early detection efforts in Mississippi.

### 4.3. Registry Trends and Regional Comparisons

Recent data from the Mississippi Cancer Registry (MCR) continue to show significant racial disparities in breast cancer outcomes within the state. According to MCR data, Black women in Mississippi continue to experience higher age-adjusted incidence and mortality rates than their White counterparts [5]. Between 2018 and 2022, Black women in Mississippi experienced an age-adjusted breast cancer mortality rate of 30 per 100,000, compared to 20 per 100,000 among White women [41]. These registry-based trends align with patterns identified in previous studies, many of which relied on older datasets, underscoring the persistent and urgent need to address racial disparities in breast cancer mortality across Mississippi. To further understand the state-specific research efforts and their relationship to breast cancer outcomes, we compared Mississippi with three neighboring states, Alabama, Louisiana, and Georgia, that share similar population characteristics and health disparities. According to the American Cancer Society, the incidence of breast cancer among women in Mississippi between 2017 and 2020 was 124.6 per 100,000 women [42]. This rate is slightly higher than in Alabama (123.3 per 100,000), but lower than in Louisiana (130.4 per 100,000) and Georgia (132.6 per 100,000) [42]. Although breast cancer incidence is generally higher among White women in the United States, rates are notably higher among Black women in Mississippi, Alabama, and Louisiana—129.7, 130.3, and 137.3 per 100,000, respectively [42]. In Georgia, the incidence rate was nearly equal between Black and White women. Mississippi also has the highest breast cancer mortality rate in the US (2016–2020), with 23.4 deaths per 100,000 women [42]. In comparison, Alabama, Louisiana, and Georgia reported slightly lower mortality rates at 20.4, 22.1, and 20.7 per 100,000, respectively. Among Black women, mortality rates remain disproportionately high: 30.4 per 100,000 in Mississippi, compared to 26.8 in Alabama, 28.1 in Louisiana, and 26.4 in Georgia [42].

### 4.4. Research Imbalance Across Southern States

Despite Mississippi having the highest breast cancer mortality rate in the nation, this review identified a limited number of studies focused on breast cancer within the state. Only 15 research articles were found over the last 25-year period. Furthermore, much of the available research was conducted decades ago. We identified only three studies focused solely on Mississippi conducted in the past 5 years, leaving critical gaps in understanding the current landscape of breast cancer in Mississippi. On the other hand, compared to Mississippi, neighboring states have a more substantial body of research on breast cancer, with more research articles published in each state in the last 5 years than Mississippi-specific publications in the last 25 years. For instance, Georgia has conducted extensive research examining breast cancer outcomes in recent years, including studies on racial disparities, social determinants of health, the impact of healthcare access, and the effectiveness of intervention programs [43]. Alabama also has a more extensive research record, including over 35 breast cancer studies published in the last five years, significantly surpassing Mississippi in terms of both volume and scope of research efforts [44]. Similarly, Louisiana has demonstrated more consistent research activity, with more than 25 publications on breast cancer in the last five years, contributing to a stronger understanding of disparities and more robust evidence to guide interventions [45]. The articles published in Alabama and Louisiana primarily focus on racial disparities, screening, early detection, access to care, treatment outcomes, and community-based interventions. This imbalance underscores the urgent need for expanded breast cancer research in Mississippi to better understand the factors driving disparities.

### 4.5. Socioeconomic and Healthcare Infrastructure Challenges

Mississippi’s broader socioeconomic and health challenges exacerbate these issues. The state ranks last in per-capita income and has the highest percentage of individuals living below the federal poverty level compared to the national average (19.6% vs. 10.5%) [40]. The poverty rate among Black residents in Mississippi is highest among all racial groups in the state and twice the rate compared to White Mississippians (31.6% vs. 12.8%) [25]. Moreover, Mississippi has a higher percentage of uninsured individuals (14%) compared to the national average (10%) [40]. The state also faces significant shortages of primary care physicians, with approximately 50% of residents living in underserved counties where there are more than 2000 people per primary care physician [40]. Furthermore, many healthcare facilities in the state require longer travel times for residents [40], creating additional barriers to care. The state’s high poverty rates, rural healthcare barriers, and limited healthcare infrastructure contribute to delayed diagnoses and lower access to high-quality care. In addition, when comparing counties in Mississippi for residential segregation, multiple counties have a high index of Black/White residential segregation [46]. Residential segregation has been found to be associated with higher concentrations of poverty, reduced access to healthcare, and poorer health outcomes [47]. These challenges are often more pronounced in rural areas with high levels of segregation, where economic hardship and long travel distances further restrict access to essential resources and healthcare services [47]. Despite this, none of the studies examined the connection between neighborhood or area deprivation and breast cancer outcomes in Mississippi.

### 4.6. Environmental and Genetic Contributors

In addition, environmental factors may also contribute to breast cancer disparities in the state. Prior studies in other regions have linked exposure to environmental chemicals to an increased risk of breast cancer [48,49]. However, there is a lack of research investigating potential environmental risk factors specific to Mississippi. Given the state’s large agricultural sector and industrial zones, future studies should explore the role of environmental chemicals in adverse breast cancer outcomes. Moreover, other key risk factors relevant to breast cancer etiology, such as behavioral, lifestyle, and hormonal influences, were not explored. These factors are well-established contributors to breast cancer development and progression, and the lack of research on these risk factors represents an important limitation of the current body of Mississippi-specific research. Additionally, none of the studies included in this review reported *BRCA1* or *BRCA2* gene expression or genetic screening for breast cancer risk. Despite only 5% to 10% of breast cancer cases being inherited, approximately 55% to 65% of individuals with a *BRCA1* gene mutation and 45% of those with a *BRCA2* gene mutation develop breast cancer by the age of 70 [50]. Future research should explore the prevalence and accessibility of genetic testing for BRCA gene mutations in Mississippi, particularly among high-risk and underserved populations.

### 4.7. Advancing Research and Bridging Gaps

As a predominantly rural state with significant differences across counties, the challenges faced by women in Mississippi may be unique; however, they have been largely unexplored in relation to breast cancer to date. The lack of recent research makes it difficult to quantify the extent of these disparities and evaluate the effectiveness of existing interventions. Addressing these knowledge gaps is crucial for guiding future research and improving healthcare strategies. More contemporary studies are needed to assess the current state of breast cancer and related disparities in Mississippi, evaluate the effectiveness of existing screening and intervention programs, and identify actionable policy solutions. Expanding research efforts in this area can help drive meaningful changes to reduce disparities, improve early detection, and ultimately lower breast cancer mortality rates in Mississippi.

### 4.8. Limitations

This review is limited by the availability of Mississippi-specific studies, highlighting the need for more state-level research on breast cancer. While the narrow geographic focus allowed for a more in-depth analysis of local challenges and disparities, it limited the generalizability of the findings. An additional limitation of this review is the exclusion of three studies for which full-text access was unavailable. Although these studies passed the initial screening based on title and abstract, the inability to retrieve the full texts prevented further quality assessment and data extraction. However, it is worth noting that these studies were published more than a decade ago and are unlikely to have significantly impacted the findings or altered the conclusions of this review. Additionally, many of the included studies were cross-sectional, limiting the ability to establish causal relationships. Future research should focus on longitudinal studies to explore trends in breast cancer outcomes over time. In addition, research is needed on neighborhood characteristics in relation to breast cancer characteristics and outcomes. Furthermore, future studies should evaluate the effectiveness of intervention programs in improving early detection and treatment adherence.

## 5. Conclusions

This systematic review underscores the urgent need to address breast cancer disparities in Mississippi, particularly among Black women and those living in rural areas. Despite leading the nation in breast cancer mortality rates, limited research has been conducted to explore this issue, with few recent studies examining the specific social, economic, and healthcare barriers contributing to poor breast cancer outcomes. To reduce breast cancer mortality and improve overall outcomes in Mississippi, more research is needed, particularly longitudinal studies, to track trends in breast cancer outcomes and their relationship to social determinants of health. Additionally, the design, implementation, and evaluation of new evidence-based strategies and programs aimed at ensuring equitable access to screening, diagnosis, and treatment for all women in Mississippi are essential. Reducing disparities and improving early detection efforts will be critical steps toward lowering breast cancer mortality rates and achieving better health outcomes across the state.

## Figures and Tables

**Table 1:** Characteristics of the included studies.

Authors, Year	Location	Study Aim	Design	Outcome of Interest	Main Findings
Higginbotham et al., 2001 [10]	Mississippi	To investigate various aspects of cancer between rural and urban localities.	Cross-sectional	Stage of breast cancer diagnosis, access to screening programs	The study found that rural individuals were more likely to be diagnosed at a later stage compared to their urban counterparts. Additionally, rural residents in Mississippi, particularly rural Black women, face significant barriers to accessing early cancer detection programs and quality medical care.
Mitra & Faruque, 2004 [11]	Mississippi	To demonstrate the relationship between environmental chemicals and breast cancer incidence in the 82 counties in Mississippi.	Cross-sectional	Breast cancer incidence rate	The study identified counties with a higher breast cancer incidence compared to the state’s median rate in 1998. Six counties—Yazoo, Copiah, George, Forrest, Stone, and Hinds—had incidence rates 40% or higher than the state average. Additionally, Noxubee, Jefferson, Jones, Perry, Scott, Chickasaw, Madison, Yalobusha, Clay, Tishomingo, and Warren also had higher rates than the state median. Harrison, Hinds, Jackson, Forrest, Rankin, Jones, Lauderdale, Perry, DeSoto, and Scott counties had the highest levels of chemical emissions, with amounts 60% or higher than the state’s median emissions. Breast cancer incidence was significantly correlated with ammonia levels, as well as the minimum and maximum emissions from facilities within these counties.
DeSantis et al., 2008 [12]	United States, including Mississippi	To investigate temporal trends in age-standardized female breast cancer mortality rates across different states and racial groups.	Cross-sectional	Breast cancer mortality rate	The study found a decline in breast cancer mortality rates among White women in all 50 states and the District of Columbia (DC), with the timing of the decline varying by state. In contrast, among Black women, breast cancer mortality rates increased in two states (Arkansas and Mississippi) out of the 37 states analyzed, remained stable in 24 states, and decreased in 11 states.
Houston, 2009 [13]	Mississippi	To highlight the persistently high breast cancer mortality rate in Mississippi, particularly among Black women, and to advocate for improved prevention and early detection.	Opinion piece (Peer-reviewed)	breast cancer mortality rate, early detection	The study advocated for healthcare professionals to collaborate with patients to modify lifestyles, encourage preventive strategies, and rigorously follow the American Cancer Society’s guidelines for early breast cancer detection.
Wilson-Anderson et al., 2013 [14]	Mississippi Delta	To provide breast health education to women in two rural counties in the Mississippi Delta.	Quasi-experimental	Breast health knowledge and screening behaviors	The study found that rural Black women were receptive to primary health education on breast cancer and showed a certain level of adherence to recommended screenings.
Keeton et al., 2014 [15]	Mississippi	To test whether race and/or geography had an impact on the stage of breast cancer at the time of diagnosis.	Cross-sectional	Stage of breast cancer diagnosis	Women living in rural Mississippi were more likely to present with advanced-stage breast cancer compared to in situ or localized breast cancer, with rates of 4% for White women and 19% for Black women. Black women residing in urban Mississippi had 25% higher odds of being diagnosed in a later stage, while rural Black women had 47% higher odds compared to their urban and rural White counterparts, respectively.
Nichols et al., 2014 [16]	Mississippi	This study aimed to assess the impact of mammography resource availability on breast cancer incidence rates, stage at initial diagnosis, mortality rates, and mortality-to-incidence ratios across Mississippi.	Cross-sectional	Breast cancer incidence rates, stage at initial diagnosis, mortality rates, and mortality-to-incidence ratios	There were no statistically significant differences in breast cancer incidence rates between Black and White women in Mississippi. However, significant disparities were observed in mammography utilization, the percentage of advanced-stage diagnoses, mortality rates, and mortality-to-incidence ratios, with Black women experiencing worse outcomes in each category. No statistically significant correlations were found between breast cancer outcomes and the availability of mammography facilities. However, mammography use was negatively correlated with the likelihood of advanced-stage diagnosis at initial presentation.
Fortune, 2015 [17]	Mississippi	This study examined the impact of the Breast and Cervical Cancer Program (BCCP) on the stage at which breast cancer is diagnosed among women in Mississippi.	Retrospective cohort	Stage of breast cancer diagnosis	Findings from this study revealed that increased screenings led to a higher number of breast cancer diagnoses. However, women enrolled in the BCCP continued to be diagnosed in later stages of the disease.
Mayfield-Johnson et al., 2016 [18]	Mississippi Delta	To increase the relatively low screening rate for African American women in the Mississippi Delta through a collaborative partnership and to decrease health disparities in breast cancer through increased awareness of self-early detection methods, leveraging resources to provide mammography screenings, and adequate follow-up with services and treatment for abnormal findings.	Cross-sectional	Breast cancer screening	In summary, 86.47% of the participants in this study were aged 40 years or older and met the clinical guidelines recommending a mammogram. However, 40.26% reported never having undergone the screening procedure, and 37.29% stated that it had been more than two years since their last mammogram, despite 93.06% having prior knowledge of mammograms. A majority of participants cited expense and access as difficulties to mammogram participation.
Fortune, 2017 [19]	Mississippi	To examine the impact of social determinants on the stage at which breast cancer is diagnosed, with a specific focus on race, income, and lack of health insurance.	Cross-sectional	Stage of breast cancer diagnosis	The study showed that Black women in Mississippi were disproportionately diagnosed in a later stage of breast cancer as opposed to an early stage. Only race and health insurance directly affected late-stage diagnosis.
Smith et al., 2017 [20]	Mississippi	To investigate the incidence of breast cancer across various health districts in Mississippi concerning healthcare disparities and accessibility.	Cross-sectional	Breast cancer incidence rate and mortality rate	The incidence and mortality rates of breast cancer among Black women in Districts VII and III are significantly higher than those in the other seven health districts of Mississippi. Additionally, Black women were diagnosed at an older age and a later stage of the disease, according to Surveillance, Epidemiology, and End Results (SEER) program staging, compared to White women. Moreover, the majority of Black women in these districts faced socioeconomic disadvantages, including higher poverty rates and lower educational attainment.
Zahnd et al., 2019 [21]	Lower Mississippi Delta Region states	To assess and characterize spatial accessibility to mammography services across eight states in the Lower Mississippi Delta Region (LMDR).	Cross-sectional	Access to mammography services	The study found clusters of low spatial access in parts of the Arkansas, Mississippi, and Tennessee Delta.
Williams et al., 2020 [22]	Mississippi	The study aimed to provide free clinical breast exams (CBEs) and breast cancer risk assessments to non-elderly African American women through a community-based breast cancer screening event.	Cross-sectional	Breast cancer screening	During the event, two healthcare providers performed clinical breast exams for 26 Black women and provided tailored risk-reduction counseling. Nearly one-third of the women screened reported never having undergone a breast cancer screening before. The authors concluded that events like this are an effective way to reach women who have never received any form of breast cancer screening.
Williams et al., 2022 [23]	Mississippi	To implement See, Test & Treat, a cancer screening and education program designed to improve access to cancer screening for underserved women in the Jackson Metropolitan Area.	Cross-sectional	Breast cancer screening	A total of 57 women received a mammogram. Participants reported that the program positively influenced their intentions to adopt healthier behaviors, with the majority stating they would perform regular self-breast exams and continue receiving routine mammograms.
Sung et al., 2023 [24]	United States, including Mississippi	To measure racial and ethnic disparities in triple-negative breast cancer (TNBC) incidence rates both between and within populations across US states.	Retrospective cohort study	Triple-negative breast cancer incidence rate	The study reported substantial racial disparities in TNBC incidence across states, showing that Black women in Mississippi, Delaware, Missouri, and Louisiana had the highest rates among all states and racial groups.

**Table 2:** Quality appraisal of included studies.

Authors	Quality Appraisal for Cross-Sectional Studies											
	Q1	Q2	Q3	Q4	Q5	Q6	Q7	Q8	Decision			
Higginbotham et al., 2001 [10]	Y	Y	Y	Y	N/A	N/A	Y	Y	Include			
Mitra & Faruque, 2004 [11]	Y	Y	Y	Y	N	N	Y	Y	Include			
DeSantis et al., 2008 [12]	Y	Y	Y	Y	Y	N	Y	Y	Include			
Keeton et al., 2014 [15]	Y	Y	Y	Y	U	N	Y	Y	Include			
Nichols et al., 2014 [16]	Y	Y	Y	Y	Y	Y	Y	Y	Include			
Mayfield-Johnson et al., 2016 [18]	Y	Y	Y	Y	Y	U	Y	Y	Include			
Fortune, 2017 [19]	Y	Y	Y	Y	U	N	Y	Y	Include			
Smith et al., 2017 [20]	Y	Y	Y	Y	N/A	N/A	Y	Y	Include			
Zahnd et al., 2019 [21]	Y	Y	Y	Y	Y	Y	Y	Y	Include			
Williams et al., 2020 [22]	Y	Y	Y	Y	N/A	N/A	Y	Y	Include			
Williams et al., 2022 [23]	Y	Y	Y	Y	U	N	Y	Y	Include			
Note. Q1: Were the criteria for inclusion in the sample clearly defined? Q2: Were the study subjects and the setting described in detail? Q3: Was the exposure measured in a valid and reliable way? Q4: Were objective, standard criteria used for measurement of the condition? Q5: Were confounding factors identified? Q6: Were strategies to deal with confounding factors stated? Q7: Were the outcomes measured in a valid and reliable way? Q8: Was appropriate statistical analysis used? Abbreviation: Y = Yes, N = No, U = Unclear, N/A = Not Applicable.												
**Authors**	**Quality Appraisal for Cohort Studies**											
	**Q1**	**Q2**	**Q3**	**Q4**	**Q5**	**Q6**	**Q7**	**Q8**	**Q9**	**Q10**	**Q11**	**Decision**
Fortune, 2015 [17]	Y	N	Y	N	N	Y	Y	Y	N/A	N/A	Y	Include
Sung et al., 2023 [24]	Y	U	Y	Y	Y	Y	Y	Y	Y	Y	Y	Include
Note. Q1: Were the two groups similar and recruited from the same population? Q2: Were the exposures measured similarly to assign people to both exposed and unexposed groups? Q3: Was the exposure measured in a valid and reliable way? Q4: Were confounding factors identified? Q5: Were strategies to deal with confounding factors stated? Q6: Were the groups/participants free of the outcome at the start of the study (or at the moment of exposure)? Q7: Were the outcomes measured in a valid and reliable way? Q8: Was the follow up time reported and sufficient to be long enough for outcomes to occur? Q9: Was follow up complete, and if not, were the reasons to loss to follow up described and explored? Q10: Were strategies to address incomplete follow up utilized? Q11: Was appropriate statistical analysis used? Abbreviation: Y = Yes, N = No, U = Unclear, N/A = Not Applicable.												
**Authors**	**Quality Appraisal for Quasi-Experimental Study**											
	**Q1**	**Q2**	**Q3**	**Q4**	**Q5**	**Q6**	**Q7**	**Q8**	**Q9**	**Decision**		
Wilson-Anderson et al., 2013 [14]	Y	N	Y	N	N	Y	Y	Y	Y	Include		
Note. Q1: Is it clear in the study what is the “cause” and what is the “effect” (i.e., there is no confusion about which variable comes first)? Q2: Was there a control group? Q3: Were participants included in any comparisons similar? Q4: Were the participants included in any comparisons receiving similar treatment/care, other than the exposure or intervention of interest? Q5: Were there multiple measurements of the outcome, both pre and post the intervention/exposure? Q6: Were the outcomes of participants included in any comparisons measured in the same way? Q7: Were outcomes measured in a reliable way? Q8: Was follow-up complete and if not, were differences between groups in terms of their follow-up adequately described and analyzed? Q9: Was appropriate statistical analysis used? Abbreviation: Y = Yes, N = No.												
**Authors**	**Quality Appraisal for Opinion Piece**											
	**Q1**	**Q2**	**Q3**	**Q4**	**Q5**	**Q6**	**Decision**					
Houston, 2009 [13]	Y	Y	Y	Y	Y	Y	Include					
Note. Q1: Is the source of the opinion clearly identified? Q2: Does the source of opinion have standing in the field of expertise? Q3: Are the interests of the relevant population the central focus of the opinion? Q4: Does the opinion demonstrate a logically defended argument to support the conclusions drawn? Q5: Is there reference to the extant literature? Q6: Is any incongruence with the literature/sources logically defended? Abbreviation: Y = Yes.												
